# Book Notices

**Published:** 1889-07

**Authors:** 


### Book Notices

CLINICAL LECTURES ON ALBUMINURIA. By Thos. Grainger Stewart, M. D., Professor of the Practice of Physic and of Clinical Medicine in the University of Edinburgh. Wm. Wood & Co., New York, 1888.

The lectures comprised in the present volume give a clear idea of all the questions involved on the subject under discussion. The book contains 350 pages, and is well worth a close study and a place in every physician's library. B.

---

A HANDBOOK OF THERAPEUTICS. By Sidney Ringer, M. D., Professor of the Principles and Practice of Medicine in University College Physician to the University College Hospital. Twelfth edition; 524 pages. Wm. Wood & Co., N. Y., 1889.

Ringer's Therapeutics is justly entitled to be called a standard work. This is really enough to say of any book. This is the twelfth edition, and it is still in active demand. B.

---

Received the ANNUAL REPORT of the BOARD OF HEALTH of the CITY OF MINNEAPOLIS, 1889. Harrison & Smith, printers, Minneapolis, Minn.

---

THE PHYSICIAN'S LEISURE LIBRARY: THE ETIOLOGY, DIAGNOSIS AND THERAPY OF TUBERCULOSIS. By Prof. Dr. H. Van Ziemssen. Pages 118. Price 25, monthly, by Geo. S. Davis, Detroit, publishers.

---

TRANSACTIONS OF THE SOUTH CAROLINA MEDICAL ASSOCIATION; 38th annual session, held at Columbia, April, 1888. Dr. C. R. Taber, Orangeburg, President; Dr. J. L. Dawson, Jr., Charleston. Correspoding Secretary.

---

TRANSACTIONS OI THE OHIO SATE MEDICAL SOCIETY, held at Columbia, June, 1888; President, Dr. P. S. Conner, Cincinnati; Secretary, Dr. G. A. Collomore, Toledo.

STATE BOARD OF HEATH OF MICHIGAN; Fifteenth annual report of the Secretary, Dr. H. B. Baker, of Lansing, 1887.

---

QUESTIONS AND ANSWERS ON THE ESSENTIALS OF SURGERY, together with a full description of the handkerchief and roller bandages. By Edward Martin, A. M., M. D., Instructor of Operative Surgery in the Unlversity of Pennsylvania, etc.

This is a nice little blue book, containing about 300 pages, put up in the form of questions and answers. The greatest amount of information can be conveyed by this plan, and since the book is intended to *cram* on, it certainly meets the indication about as well as any other of the kind. B.

---

EXPLORATION OF THE CHEST IN HEALTH AND DISEASE. By Stephen Smith Burt, M. D., Professor of Clinical Medicine and Physical Diagnosis in the New York Post Graduate School and Hospital. N. Y., D. Appleton & Co., 1889.

This little book of 200 pages is very handy and practical. It covers the ground succinctly, giving the busy physician a clear conception of his subject without loss of time. It is a good book.

B.

---

THE VEST-POCKET ANATOMIST (founded upon "Gray"). By C. Henri Leonard, A. M., M. D., Professor of the Medical and Surgical Diseases of Women and Clinical Gynecology in the Detroit College of Medicine, etc. 193 illustrations. 14th revised edition, containing dissection hints and visceral anatomy. Publishers: The Illustrated Medical Journal Co., Detroit. Pages, 297; price, $1.

An abbridged work on anatomy, such as this, does more harm than good. The student depends upon it to the neglect of the dissecting room, the only source from which he can obtain the proper foundation. This work has reached a larger sale, however, than any other of the kind known to the writer.

---

TTANSACTIONS OF THE MEDICAL SOCIETY OF NEW YORK for 1885, 1886, 1887 and 1888; President, Dr. S. B. Ward, Albany; Secretary, Dr. W. M. Smith, Syracuse.

TRANSACTIONS OF THE MISSISSIPPI STATE MEDICAL ASSOCIATION, 1888; President, Dr. Luther Sexton, Wesson; Correiponding Secretary, Dr. W. A. Galloway, Jackson.

---

A NEW MEDICAL JOURNAL.—*The Kansas Medical Journal*, edited by Drs. W. L. Schenck, Osage City; J. E. Minney, Topepa, and S. G. Stewart, Topeka. Issued from Topeka, monthly. It is a fine looking journal,--printed well and edited well. Success.

---

TRANSACTIONS OF THE MEDICAL ASSOCIATION OF THE STATE OF MISSOURI; 31st annual session, held at Kansas City, April, 1888; President, Dr. A. W. McAlester, Columbia, Corresponding Secretary, Dr. L. J. Mathews, of Carthage.

